# Factors affecting foveal avascular zone in healthy eyes: An examination using swept-source optical coherence tomography angiography

**DOI:** 10.1371/journal.pone.0188572

**Published:** 2017-11-27

**Authors:** Atsushi Fujiwara, Yuki Morizane, Mio Hosokawa, Shuhei Kimura, Yusuke Shiode, Masayuki Hirano, Shinichiro Doi, Shinji Toshima, Kosuke Takahashi, Mika Hosogi, Fumio Shiraga

**Affiliations:** Department of Ophthalmology, Okayama University Graduate School of Medicine, Dentistry and Pharmaceutical Sciences, Okayama, Japan; Universidade do Minho, PORTUGAL

## Abstract

**Objective:**

To examine factors affecting foveal avascular zone (FAZ) area in healthy eyes using swept-source optical coherence tomography angiography (OCTA).

**Methods:**

This prospective, cross-sectional study included 144 eyes of 144 individuals (77 women, 67 men) with a best corrected visual acuity of at least 20/20 and no history of ocular disorders. The area of the superficial FAZ was assessed using OCTA. Age, gender, central retinal thickness (CRT), retinal vascular density, refractive error, and axial length were examined to determine associations with FAZ area.

**Results:**

The mean age of the subjects was 42.1 ± 20.2 years (range: 10–79 years). The mean FAZ area was 0.32 ± 0.11 mm^2^, while the mean retinal vascular density was 35.53 ± 0.92%. Multivariate regression analysis was performed using FAZ area as the dependent variable and age, gender, CRT, retinal vascular density, refractive error, and axial length as independent variables. The results of this analysis demonstrate that CRT and retinal vascular density were significantly associated with FAZ area in our sample (P < 0.001, R^2^ = 0.425). Age, gender, refractive error, and axial length were not significantly correlated with FAZ area, while CRT and retinal vascular density were negatively correlated with FAZ area (CRT: P < 0.001, R^2^ = 0.356; retinal vascular density: P < 0.001, R^2^ = 0.189).

**Conclusions:**

OCTA results suggest that CRT and retinal vascular density negatively affect FAZ area in healthy eyes.

## Introduction

Optical coherence tomography angiography (OCTA) is a non-invasive imaging system with microvasculature visualization capability superior to that of fluorescein angiography.[1] Dye-free OCTA is not affected by multiple scattering caused by fluorescein fluorescence arising from nerve fiber bundles. As such, higher resolution images of the radial peripapillary capillary network and the deep capillary network can be obtained by OCTA compared to FA.[1] Recently, OCTA has been used to visualize retinal microvasculature, particularly within the foveal avascular zone (FAZ)[2–4]. The FAZ is a capillary-free area in the central macula and is a specialized region of the human retina that is in proximity to the region of highest cone photoreceptor density and oxygen consumption.[5–7] Variations in the area of the FAZ may be associated with visual function and have been shown to be of both diagnostic and prognostic value in various retinal diseases.[7–15] For example, in cases of diabetic retinopathy and retinal vein occlusion, FAZ area is expanded compared to healthy eyes, and this expansion has been reported to be related to visual function.[7–15] Moreover, diabetic eyes are known to exhibit macular microcirculation impairment even before the onset of retinopathy, and FAZ area assessment has been reported to allow early detection of diabetic retinopathy.[9]

To determine the relationship between variation in FAZ area and retinal disease, it is essential to first understand the variation in FAZ area in healthy individuals. Previous studies have reported significant variation in FAZ area in healthy eyes[4,16–22], and it is therefore important to ascertain the demographic and ocular factors that may underlie this variation[4]. Previous studies have examined the relationship between FAZ area and retinal vascular density, age, gender, race, axial length, refractive error, and macular shape, which includes central retinal thickness (CRT), inner retinal layer thickness, and foveal pit shape[2–4,18,23,24]. However, these factors have only been examined in isolation; that is, no study has examined all of these factors together. Furthermore, these previous studies contained bias in the age groups of the eyes examined. Because age, CRT, and retinal vascular density are associated with each other, it is possible that the relationships between FAZ area and these factors have not been examined accurately.[2] Therefore, there is a need for a study which expands the age groups of included subjects, equalizes the number of eyes in each age group, and examines the relationships of these factors to FAZ area within the same cohort.

In the present study, we used swept-source OCTA to quantify FAZ area and examined the relative contributions of age, gender, CRT, retinal vascular density, refractive error, and axial length to FAZ area by multivariate analysis.

## Materials and methods

### Subjects

This prospective cross-sectional study examined 144 eyes in 144 individuals aged 10–79 years. All of the subjects were Japanese. The inclusion criteria consisted of a best corrected visual acuity of 20/20 or better and a spherical equivalent of −6 D and above and +6 D and below. Patients were excluded if they presented with any history or clinical evidence of glaucoma, strabismus, or retinal disease such as diabetic retinopathy, age-related macular degeneration, or corneal degeneration. Additionally, patients with a prior history of ocular surgery or laser photocoagulation were excluded.

For all patients, we examined the right eye and performed tests under non-mydriatic condition. All of the investigative procedures conformed to the tenets of the Declaration of Helsinki. This study was approved by the Ethics Committee of Okayama University Hospital, Okayama, Japan. We explained the nature and possible consequences of the study, and written informed consent was obtained from all adult subjects and from the guardians of all subjects who were minors.

### Optical coherence tomography angiography

We captured OCTA images with a DRI OCT-1 Atlantis (Topcon Corporation, Tokyo, Japan), which uses swept-source OCT technology and has a long-wavelength light source of approximately 1050 nm and a scanning speed of 100,000 A-scans per second.[25,26] The retinal region of interest was the center of the fovea (a 3.0 × 3.0 mm area), and the A-scan density was 320 lines (horizontal) × 320 lines (vertical). Refractive error (spherical and astigmatic errors) and corneal curvature radius were measured with an RC-5000 (Tomey Corporation, Nagoya, Japan), and axial length was measured on the same day with an optical biometer (OA-2000, Tomey Corporation, Nagoya, Japan). The resulting data were entered into the DRI OCT-1 Atlantis in advance and were used to correct for magnification errors that arise from differences in axial length. Software based on Littmann’s method was installed onto the DRI OCT-1 Atlantis, and adjustments for magnification effects were thus performed automatically to calculate FAZ area, CRT, and retinal vascular density after entering values for axial length, refractive power, and corneal curvature radius.[26,27]

### Measurement of FAZ area

**Fig 1:**
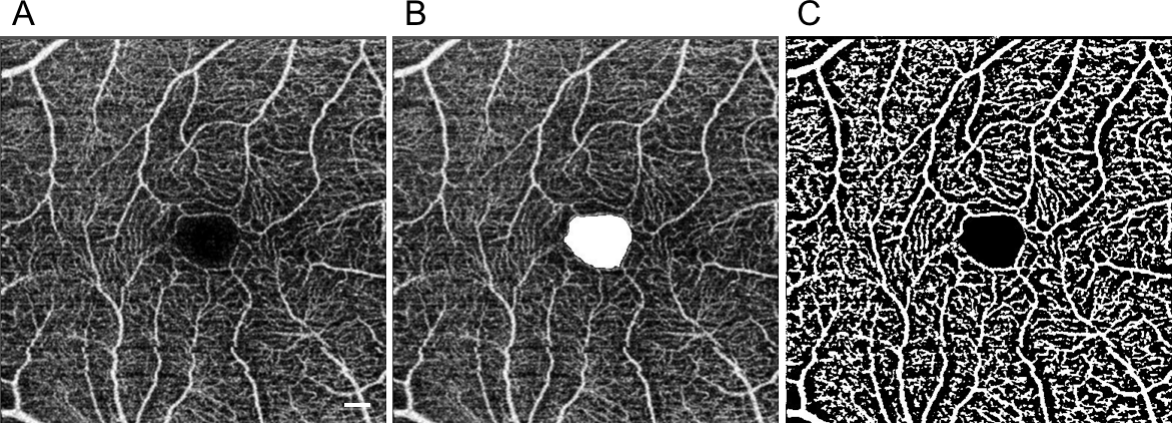
Analysis of optical coherence tomography angiography image. Typical optical coherence tomography angiography (OCTA) images. Bar = 200 μm (A) Superficial capillary plexus en face image obtained with OCTA. (B) The foveal avascular zone (FAZ) is displayed in white; this image is based on the superficial capillary plexus en face image in A. (C) The superficial capillary plexus en face image from A binarized with ImageJ using Niblack’s method. Images such as C were used to measure retinal vascular density.

FAZ area was measured based on superficial capillary plexus en face imaging (Fig 1A) using EnView (Ver. 1.2.0, Topcon Corporation) analysis software, which was installed on the DRI OCT-1 Atlantis. The superficial capillary plexus en face image was segmented with an inner boundary at 2.6 μm beneath the internal limiting membrane and an outer boundary at 15.6 μm beneath the inner plexiform layer [3,28]. As shown in Fig 1B, the borderline of the FAZ was traced manually, and the area of the FAZ was then calculated by the Enview analysis software.[29]

### Calculation of retinal vascular density

To quantify the retinal vascular density, the superficial capillary plexus en face image (Fig 1A) was binarized according to Niblack’s method with ImageJ software (version 1.48; National Institutes of Health, Bethesda, MD, USA; Fig 1C).[26,30,31] The white area in each resulting image was defined as the retinal vascular area, and the pixel number was quantified with ImageJ software (Fig 1C). Retinal vascular density was calculated by dividing the retinal vascular area by the value obtained from subtracting the FAZ area from the area of the region of interest (3 × 3 mm, 263169 pixels).

### Measurement of central retinal thickness

Images of the center of the fovea had a 12.0 x 9.0 mm area and were taken with the DRI OCT-1 Atlantis. The A-scan density was 512 lines (horizontal) × 256 lines (vertical). The retinal thickness in the central area of the ETDRS circle (1 mm in diameter) was measured automatically by the analysis software installed on the DRI OCT-1 Atlantis, and this measurement was used as the CRT value. [32,33] Measurements were taken automatically using this analysis software to avoid the subjectivity of a human measurer and to allow for the rapid analysis of large amounts of data.

### Statistical analysis

All statistical analyses were performed using SPSS version 22.0 (IBM Corp, Armonk, NY, USA). Variable normality was confirmed using the Shapiro-Wilk test for all variables. To assess the variables affecting FAZ area, a multivariate regression analysis was performed with FAZ area as the dependent variable and age, gender, CRT, retinal vascular density, refractive error, and axial length as independent variables. In addition, a univariate regression analysis was conducted to assess the relationship between FAZ area and age, CRT, retinal vascular density, refractive error, and axial length. Student’s t-tests were used to assess the effects of gender on FAZ area, CRT, and retinal vascular density. A *P*-value of <0.05 was considered to be statistically significant.

## Results

### Demographic data

Subjects’ demographic data are shown in Table 1. The mean age of our sample was 42.1 ± 20.2 years (10–79 years). Seventy-seven subjects (53.5%) were women. The mean FAZ area of our sample was 0.32 ± 0.11 mm^2^ (0.09–0.63 mm^2^), the mean CRT was 235.3 ± 19.5 μm (164.0–286.0 μm), the mean retinal vascular density was 35.53 ± 0.92% (33.2–39.2%), the mean refractive error was -1.30 ± 2.33 D (−6.00 –+5.00 D), and the mean axial length was 24.0 ± 1.3 mm (20.4–27.6 mm). All relevant data are available in S1 Table.

**Table 1:** Demographic data by age group.

Age group	Age (years)	N (eyes)	Gender (F / M)	Foveal avascular zone area (mm^2^)	Central retinal thickness (μm)	Retinal vascular density (%)	Refractive error (D)	Axial length (mm)
10–19	13.8 ± 3.0	26	11 / 15	0.33 ± 0.11	227.4 ± 20.0	35.79 ± 0.90	-0.59 ± 2.54	23.9 ± 1.4
20–29	25.5 ± 2.2	16	6 / 10	0.30 ± 0.11	232.9 ± 19.5	35.62 ± 0.69	-2.63 ± 1.99	24.6 ± 1.4
30–39	33.9 ± 3.0	27	16 / 11	0.32 ± 0.10	239.0 ± 21.1	35.87 ± 0.57	-2.95 ± 2.50	24.6 ± 1.6
40–49	43.6 ± 3.1	21	15 / 6	0.27 ± 0.09	244.6 ± 17.8	35.83 ± 1.06	-1.76 ± 1.27	24.0 ± 1.0
50–59	53.8 ± 3.1	17	10 / 7	0.34 ± 0.10	237.8 ± 15.0	35.23 ± 0.78	-0.88 ± 2.00	23.7 ± 1.0
60–69	64.6 ± 2.3	23	14 / 9	0.35 ± 0.12	236.0 ± 15.8	35.23 ± 0.96	-0.27 ± 1.84	23.5 ± 1.1
70–79	76.6 ± 2.9	14	5 / 9	0.36 ± 0.09	227.8 ± 19.1	34.63 ± 0.72	0.56 ± 0.92	23.3 ± 0.8
Total	42.1 ± 20.2	147	77 / 67	0.32 ± 0.11	235.3 ± 19.5	35.53 ± 0.92	-1.30 ± 2.33	24.0 ± 1.3

All values are given as mean ± standard deviation except number of eyes and sex. F, female; M, male

### Multivariate regression analysis

We performed multivariate regression analysis using FAZ area as the dependent variable and age, gender, CRT, retinal vascular density, refractive error, and axial length as independent variables. The results showed that CRT and retinal vascular density were significantly associated with FAZ area in our sample (*P* < 0.001, y = 2.197 − 0.003×CRT − 0.034×retinal vascular density, R^2^ = 0.425, Table 2).

**Table 2:** Multivariable regression analysis for the foveal avascular zone area.

				95% CI	
Independent variable	*β*	Standard error	*P* value	Minimum	Maximum
Constant	2.197	0.269	< 0.001	1.666	2.729
Age			0.110		
Gender			0.116		
Central retinal thickness	-0.003	0.001	< 0.001	-0.004	-0.002
Retinal vascular density	-0.034	0.008	< 0.001	-0.049	-0.018
Refractive error			0.410		
Axial length			0.119		

*β*: Partial regression coefficient, N = 147, R^2^ = 0.425. CI, confidence interval

### Univariate regression analysis

**Fig 2:**
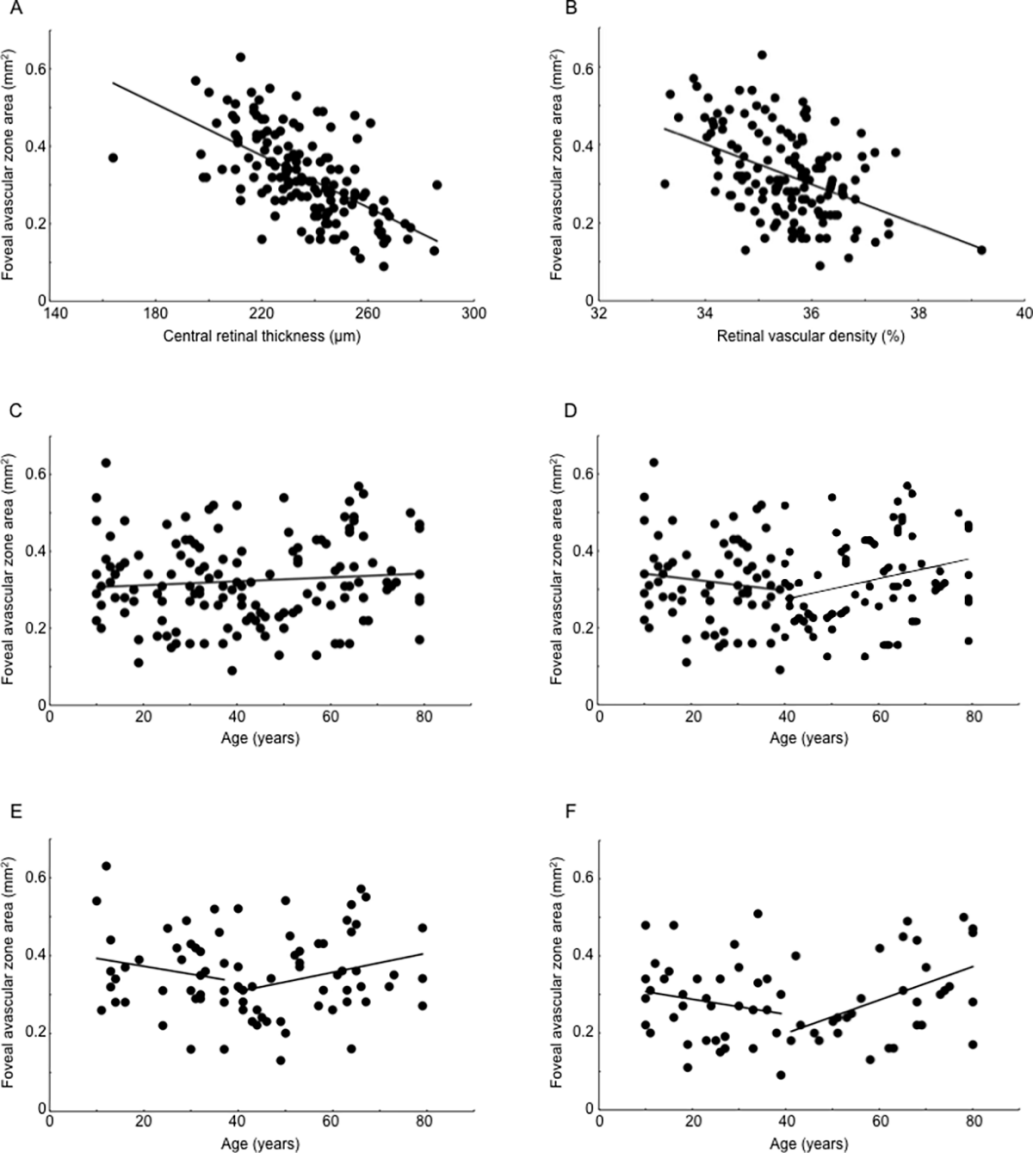
Association of foveal avascular zone area with central retinal thickness, retinal vascular density, age and gender in healthy eyes. (A) Central retinal thickness (CRT) was negatively correlated with foveal avascular zone (FAZ) area (*P* < 0.001, y = −0.003x + 1.112, R^2^ = 0.356). (B) Retinal vascular density was negatively correlated with FAZ area (*P* < 0.001, y = −0.052x + 2.157, R^2^ = 0.189). (C) Age was not significantly correlated with FAZ area (*P* = 0.261, y = 0.001x + 0.302, R^2^ = 0.188). (D) For subjects younger than 40 years, age was not significantly correlated with FAZ area (*P* = 0.312, y = −0.002x + 0.356, R^2^ = 0.178). However, for subjects aged 40 years or older, age was positively correlated with FAZ area (*P* = 0.001, y = 0.001x + 0.250, R^2^ = 0.258). (E, F) Relationship between age and FAZ area by gender. (E) Among women younger than 40 years, age was not significantly correlated with FAZ area (*P* = 0.318, y = −0.002x + 0.414, R^2^ = 0.052). However, among women 40 years or older, age was positively correlated with FAZ area (*P* = 0.031, y = 0.003x + 0.208, R^2^ = 0.167). (F) Among men younger than 40 years, age was not significantly correlated with FAZ area (*P* = 0.295, y = −0.002x + 0.327, R^2^ = 0.062). However, among men 40 years or older, age was positively correlated with FAZ area (*P* = 0.006, y = 0.004x + 0.031, R^2^ = 0.223).

The results of the univariate regression analyses performed for FAZ area and each variable are displayed in Table 3. CRT and retinal vascular density were each found to be negatively correlated with FAZ area (CRT: *P* < 0.001, R^2^ = 0.356; retinal vascular density: *P* < 0.001, R^2^ = 0.189; Fig 2A and 2B).

**Table 3:** Univariate analysis predicting foveal avascular zone area.

Model	Constant	B	*P* value	R^2^
Age	0.001	0.302	0.261	0.188
Central retinal thickness	-0.003	1.112	< 0.001	0.356
Retinal vascular density	−0.052	2.157	< 0.001	0.189
Refractive error	0.001	0.324	0.923	0.162
Axial length	−0.004	0.414	0.559	0.102

B: Unstandardized coefficients

We found no significant correlation between age and FAZ area (*P* = 0.261, R^2^ = 0.188, Fig 2C). Because a previous study reported that FAZ area expands significantly after the age of 40[16], we conducted another examination using the age of 40 as a cutoff point. Among subjects younger than 40, age was not significantly correlated with FAZ area (*P* = 0.312, R^2^ = 0.178, Fig 2D). However, among subjects aged 40 years or older, age was positively correlated with FAZ area (*P* = 0.001, R^2^ = 0.258, Fig 2D).

An examination of the relationship between FAZ area and age by gender did not reveal a significant correlation between FAZ area and age among women or men across all ages or among subjects younger than 40 (women overall: *P* = 0.318, R^2^ = 0.062; men overall; *P* = 0.231, R^2^ = 0.200; women aged <40 years; *P* = 0.318, R^2^ = 0.052; men aged <40 years: *P* = 0.295, R^2^ = 0.062; Fig 2E and 2F). However, among both women and men aged 40 years or older, age was positively correlated with FAZ area (women aged ≥40 years: *P* = 0.031, R^2^ = 0.167; men aged ≥40 years: *P* = 0.006, R^2^ = 0.223; Fig 2E and 2F). No significant difference in FAZ area was observed between genders (women: 0.35 ± 0.10 mm^2^, men: 0.29 ± 0.11 mm^2^, *P* = 0.747).

Refractive error and axial length were not significantly correlated with FAZ area (refractive error: *P* = 0.923, R^2^ = 0.162; axial length: *P* = 0.559, R^2^ = 0.102; S1A and S1B Fig).

The associations of CRT with age, gender, refractive error, axial length, and retinal vascular density are displayed in S2 Fig, and the relationships between retinal vascular density and age, gender, refractive error, and axial length are displayed in S3 Fig.

## Discussion

To our knowledge, the present study is the first to examine the relationships between FAZ area and CRT, retinal vascular density, age, gender, axial length, and refractive error in the same sample of healthy subjects. Multivariate analysis revealed that CRT and retinal vascular density were significantly associated with FAZ area in our sample of healthy subjects (Table 2). In contrast, FAZ area was not found to be significantly associated with age, gender, axial length, or refractive error. Prior studies have investigated the relationship between FAZ area and CRT alone as well as the relationship between FAZ area and retinal vascular density alone. These studies have reported that CRT is negatively correlated with FAZ area[3,4,18,22], while retinal vascular density has been found to be unrelated to FAZ area[2,20,24]. Our results support the results of previous studies on the association of FAZ area and CRT, and we also report for the first time that FAZ area is associated with retinal vascular density.

Both CRT and retinal vascular density were found to be related to FAZ area, but a comparison of these effects suggests that CRT has a greater relationship with FAZ area than does retinal vascular density. There are two possible reasons for this finding. First, while both CRT and retinal vascular density demonstrated highly significant correlations with FAZ area by univariate analysis, CRT demonstrated a stronger correlation (Fig 2A and 2B; CRT: *P* < 0.001, R^2^ = 0.356; retinal vascular density: *P* < 0.001, R^2^ = 0.189). A strong correlation between FAZ area and CRT has been reported in past studies using healthy eyes[3,4,18,19,22], and the results of the present study are consistent with these results. Our results are also consistent with Hendrickson’s developmental model of the primate fovea, which postulates that the formation of the FAZ, which precedes formation of the foveal pit, is one of the mechanical factors that determines foveal differentiation[34–38]. Second, compared to CRT, retinal vascular density demonstrated little variation between individuals (S2A Fig, S3A Fig and Table 1) and thus appeared to have little effect on FAZ area (Fig 2A and 2B). These results suggest that when comparing FAZ area between individuals in clinical research, adjusting for CRT is more important than adjusting for retinal vascular density.

CRT remains stable throughout life, but the ganglion cell layer and the retinal nerve fiber layer each thin as the eye ages[39]. Indeed, in the current study, no significant correlation was observed between age and CRT (S2A Fig). This finding suggests that the effects of CRT on FAZ area do not require much consideration when examining age-related changes in FAZ area, but these effects do require attention when comparing FAZ area between individuals. Although CRT was the only parameter related to foveal shape that was examined in the present study, past studies have reported the importance of parameters other than CRT as indicators of foveal shape[19,22]. Further study will be necessary to understand the relationships between FAZ area and other parameters associated with foveal shape.

Of all the factors potentially affecting FAZ area that were examined in the present study, age is likely the most controversial factor. Studies by Laatikainen et al[16], Yu et al[2] and Iafe et al[40] selected healthy eyes as study targets using the same criteria as the present study, each reported that as age advances, FAZ area expands, parafoveal blood flow decreases, and capillaries become occluded. However, Samara et al[3], Gadde et al[24], and Tan et al[4] also investigated healthy eyes each of these studies reported that age and FAZ area are not significantly correlated. Similarly, the results of both the multivariate and the univariate analyses in the present study did not show a significant relationship between age and FAZ area in healthy eyes (Table 2 and Fig 2A). Laatikainen et al[16] measured FAZ area using fundus fluorescein angiography and found that subjects aged 40 years or older demonstrated a stronger correlation between FAZ area and age compared to those aged less than 40 years. Therefore, we conducted a similar analysis in the present study and observed a significant correlation between age and FAZ area in the eyes of the 78 patients aged 40 years and older by univariate analysis (Fig 2D). This trend was observed among both men and women. However, when we performed multivariate analysis including only subjects aged 40 years and older, age was not found to be associated with FAZ area. These inconsistencies may be explained by differences in the methods of visualization and measurement of FAZ area, differences in the number of samples per age group, and differences in the ethnicities of the target subjects. Further studies with more subjects are needed in order to determine the relationship between age and FAZ area.

Using an image to derive the true size of any object on the fundus requires the measurements of several parameters that contribute to the image, including axial length and the curvature and thickness of the cornea[27]. If the corrections derived from these data are not used, magnification errors arise and FAZ area, CRT, and retinal vascular density cannot be accurately measured. As such, in the present study, we used analysis software installed in the swept-source OCT in order to quantify FAZ area, CRT, and vascular density. In doing so, each patient’s axial length, refractive error, and corneal curvature radius was entered into the swept-source OCT in advance in order to allow adjustments to be made.[26] However, it is unclear whether past studies performed similar adjustments. To our knowledge, the Optovue RTVue XR 100 Avanti, version 2014.2.0.13 (Optovue Inc, Fremont, CA, USA), as one example, does not have a function that performs such adjustments[40]. We believe that the above adjustments should be performed in order to accurately quantify and compare FAZ area, CRT, and vascular density.

The present study has several important limitations. First, we did not examine FAZ area or retinal vascular density in the deep retinal layers. Retinal vascular density is greater in the deep layers compared to the superficial layers, and the FAZ in the deep layers does not form a clear-cut ring as it does in the superficial layers[3,41]. Furthermore, the deep layers are affected by projection artifacts[42,43]. Therefore it was difficult to accurately quantify FAZ area and retinal vascular density in the deep layers using the OCT resolution available for the present study. Second, the present study analyzed data only from Japanese individuals; thus, we did not examine the effects of race. Past studies have reported racial differences in foveal structure[44,45], and further study is necessary to determine whether the results of the present study apply to other races. In conclusion, the present study demonstrates that CRT and retinal vascular density are associated with FAZ area in healthy eyes. Therefore, these factors must be considered in order to accurately assess the relationship between FAZ area and retinal disease.

## Supporting information

S1 Table Demographic data of all subjects. (XLSX)

S1 Fig Associations of foveal avascular zone area with refractive error and axial length in healthy eyes. (A) Refractive error was not significantly correlated with FAZ area (*P* = 0.923, y = 0.001x + 0.324, R^2^ = 0.162). (B) Axial length was not significantly correlated with FAZ area (*P* = 0.559, y = −0.004x + 0.414, R^2^ = 0.102).
(TIF)

S2 Fig Associations of central retinal thickness with age, gender, retinal vascular density, refractive error, and axial length in healthy eyes. (A) Age was not significantly correlated with central retinal thickness (CRT) (*P* = 0.256, y = 0.092x + 231.77, R^2^ = 0.008). (B, C) Relationship between age and CRT by gender. (B) Among women, age was not significantly correlated with CRT (*P* = 0.046, y = 0.222x + 221.19, R^2^ = 0.052). (C) Among men, age was not significantly correlated with CRT (*P* = 0.934, y = 0.001x + 24.97, R^2^ = 0.001). CRT was significantly higher in men compared to women (women: 230.8 ± 19.0 μm, men: 241.3 ± 19.0 μm, *P* = 0.001). (D) Retinal vascular density was positively correlated with CRT (*P* < 0.001, y = 5.502x + 40.30, R^2^ = 0.168). (E) Refractive error was not significantly correlated with CRT (*P* = 0.292, y = −0.740x + 234.68, R^2^ = 0.007). (F) Axial length was not significantly correlated with CRT (*P* = 0.285, y = 0.721x + 218.04, R^2^ = 0.002).
(TIF)

S3 Fig Associations of retinal vascular density with age, gender, refractive error, and axial length in healthy eyes. (A) Age was negatively correlated with retinal vascular density (*P* < 0.001, y = −0.016x + 36.21, R^2^ = 0.326). (B, C) Association between age and retinal vascular density by gender. (B) Among women, age was negatively correlated with retinal vascular density (*P* < 0.001, y = −0.016x + 36.21, R^2^ = 0.146). (C) Among men, age was negatively correlated with retinal vascular density (*P* < 0.001, y = −0.016x + 36.37, R^2^ = 0.246). The retinal vascular density was significantly higher in the men in our sample compared to the women (women: 35.33 ± 0.97%, men: 35.72 ± 0.82%, *P* = 0.013). (D) Refractive error was not significantly correlated with retinal vascular density (*P* = 0.473, y = −0.025x + 35.47, R^2^ = 0.067). (E) Axial length was not significantly correlated with retinal vascular density (*P* = 0.312, y = 0.067x + 33.89, R^2^ = 0.040).
(TIF)
