# Supplementary figures and images for: Factors affecting foveal avascular zone in healthy eyes: An examination using swept-source optical coherence tomography angiography
Source: PLoS One. 2017 Nov 27;12(11):e0188572. doi: 10.1371/journal.pone.0188572 (PMC5703551; doi:10.1371/journal.pone.0188572)

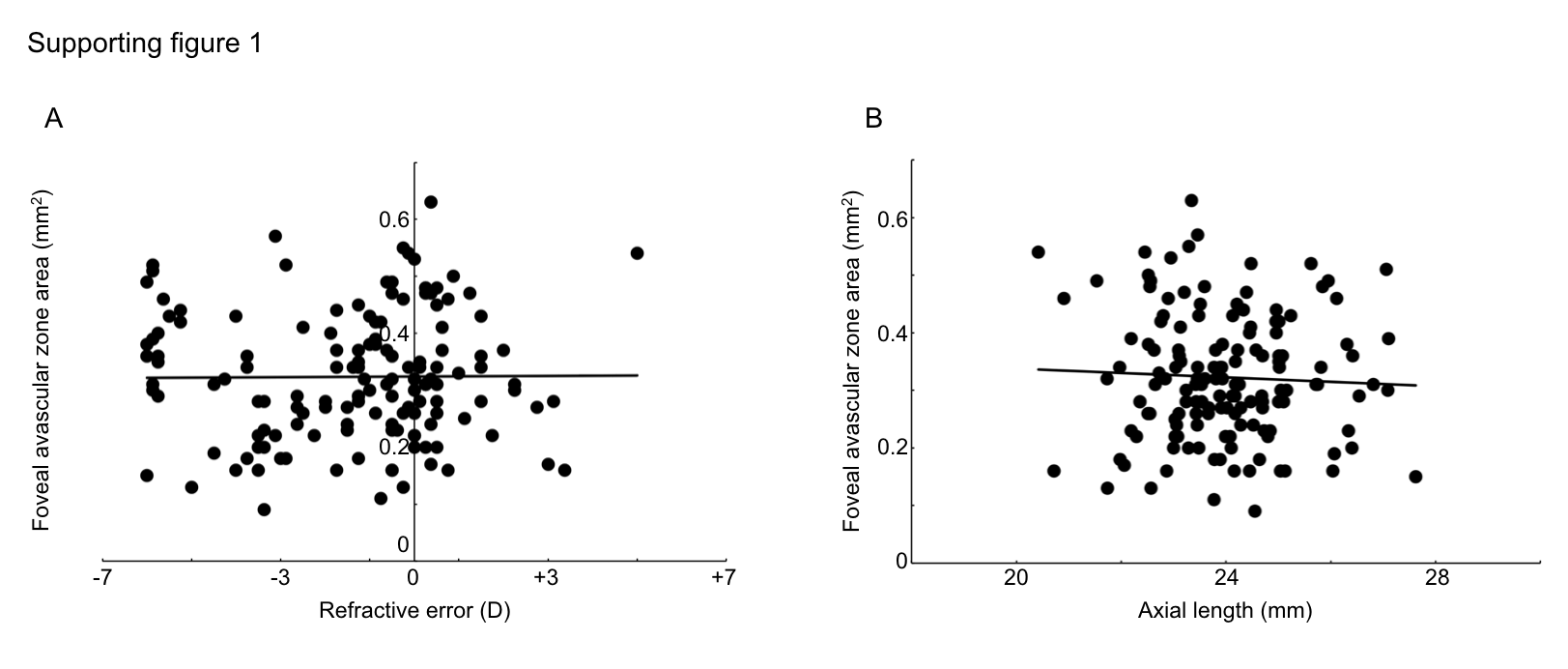

Supplement: S1 Fig — (A) Refractive error was not significantly correlated with FAZ area (P = 0.923, y = 0.001x + 0.324, R2 = 0.162). (B) Axial length was not significantly correlated with FAZ area (P = 0.559, y = −0.004x + 0.414, R2 = 0.102). (TIF) [file pone.0188572.s002.tif]

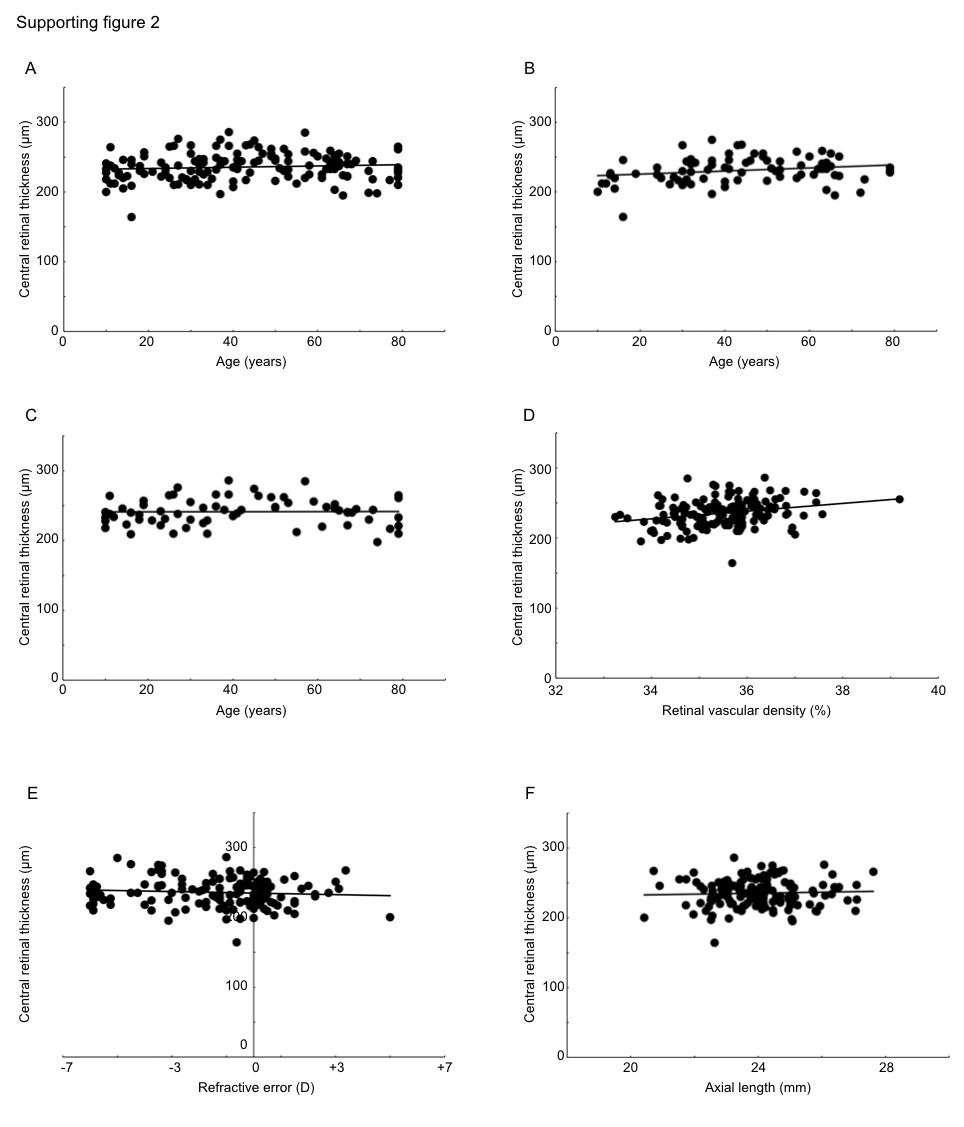

Supplement: S2 Fig — (A) Age was not significantly correlated with central retinal thickness (CRT) (P = 0.256, y = 0.092x + 231.77, R2 = 0.008). (B, C) Relationship between age and CRT by gender. (B) Among women, age was not significantly correlated with CRT (P = 0.046, y = 0.222x + 221.19, R2 = 0.052). (C) Among men, age was not significantly correlated with CRT (P = 0.934, y = 0.001x + 24.97, R2 = 0.001). CRT was significantly higher in men compared to women (women: 230.8 ± 19.0 μm, men: 241.3 ± 19.0 μm, P = 0.001). (D) Retinal vascular density was positively correlated with CRT (P < 0.001, y = 5.502x + 40.30, R2 = 0.168). (E) Refractive error was not significantly correlated with CRT (P = 0.292, y = −0.740x + 234.68, R2 = 0.007). (F) Axial length was not significantly correlated with CRT (P = 0.285, y = 0.721x + 218.04, R2 = 0.002). (TIF) [file pone.0188572.s003.tif]

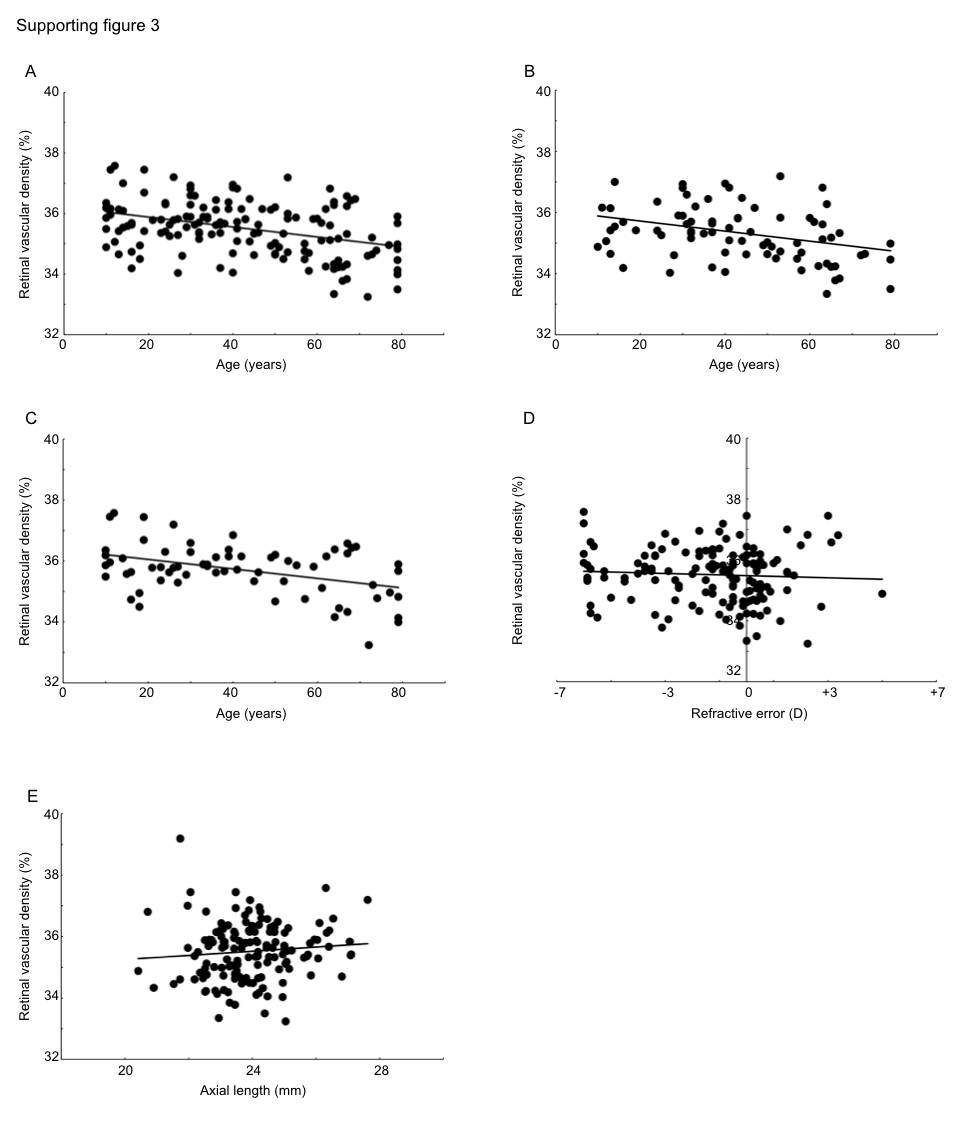

Supplement: S3 Fig — (A) Age was negatively correlated with retinal vascular density (P < 0.001, y = −0.016x + 36.21, R2 = 0.326). (B, C) Association between age and retinal vascular density by gender. (B) Among women, age was negatively correlated with retinal vascular density (P < 0.001, y = −0.016x + 36.21, R2 = 0.146). (C) Among men, age was negatively correlated with retinal vascular density (P < 0.001, y = −0.016x + 36.37, R2 = 0.246). The retinal vascular density was significantly higher in the men in our sample compared to the women (women: 35.33 ± 0.97%, men: 35.72 ± 0.82%, P = 0.013). (D) Refractive error was not significantly correlated with retinal vascular density (P = 0.473, y = −0.025x + 35.47, R2 = 0.067). (E) Axial length was not significantly correlated with retinal vascular density (P = 0.312, y = 0.067x + 33.89, R2 = 0.040). (TIF) [file pone.0188572.s004.tif]
